# Excessive daytime sleepiness and falls among older men and women: cross-sectional examination of a population-based sample

**DOI:** 10.1186/s12877-015-0068-2

**Published:** 2015-07-05

**Authors:** Amie C. Hayley, Lana J. Williams, Gerard A. Kennedy, Kara L. Holloway, Michael Berk, Sharon L. Brennan-Olsen, Julie A. Pasco

**Affiliations:** 1 IMPACT SRC, School of Medicine, Deakin University, Barwon Health, PO Box 281, Geelong, Australia; 2 Centre for Human Psychopharmacology, Swinburne University of Technology, Hawthorn, Australia; 3 Department of Psychiatry, The University of Melbourne, Level 1 North, Main Block, Royal Melbourne Hospital, Parkville, Australia; 4 School of Psychology, Counselling & Psychotherapy, Cairnmillar Institute, 993 Burke Road, Camberwell, Australia; 5 Institute for Breathing and Sleep, Bowen Centre, Austin Health, PO Box 5555, Heidelberg, Melbourne Australia; 6 Orygen, the National Centre of Excellence for Youth Mental Health, 35 Poplar Rd, Parkville, Melbourne Australia; 7 Florey Institute for Neuroscience and Mental Health, 30 Royal Parade, Parkville, Melbourne Australia; 8 NorthWest Academic Centre, Department of Medicine, The University of Melbourne, CHRE Building, Level 3 East, Sunshine Hospital, 176 Furlong Road, St Albans, Melbourne Australia

**Keywords:** Excessive daytime sleepiness, Falls, Older adults, Epidemiology, Population, Elderly

## Abstract

**Background:**

Excessive daytime sleepiness (EDS) has been associated with an increased risk for falls among clinical samples of older adults. However, there is little detailed information among population-representative samples. The current study aimed to assess the relationship between EDS and falls among a cohort of population-based older adults.

**Methods:**

This study assessed 367 women aged 60-93years (median 72, interquartile range 65-79) and 451 men aged 60-92years (median 73, interquartile range 66-80) who participated in the Geelong Osteoporosis Study between the years 2001 and 2008. Falls during the prior year were documented via self-report, and for men, falls risk score was obtained using an Elderly Fall Screening Test (EFST). Sleepiness was assessed using the Epworth Sleepiness Scale (ESS), and scores of ≥ 10 indicated EDS. Differences among those with and without EDS in regard to falls were tested using logistic regression models.

**Results:**

Among women, 50 (13.6 %) individuals reported EDS. Women with EDS were more likely to report a fall, and were more likely to report the fall occurring outside. EDS was similarly associated with an increased risk of a fall following adjustment for use of a walking aid, cases of nocturia and antidepressant medication use (adjusted OR = 2.54, 95 % CI 1.24-5.21). Multivariate modelling revealed antidepressant use (current) as an effect modifier (*p* < .001 for the interaction term). After stratifying the data by antidepressant medication use, the association between EDS and falls was sustained following adjustment for nocturia among antidepressant non-users (adjusted OR = 2.63, 95 % CI 1.31-5.30). Among men, 72 (16.0 %) individuals reported EDS. No differences were detected for men with and without EDS in regard to reported falls, and a trend towards significance was noted between EDS and a high falls risk as assessed by the EFST (p = 0.06), however, age explained this relationship (age adjusted OR = 2.20, 95 % CI 1.03-1.10).

**Conclusions:**

For women, EDS is independently associated with at least one fall during the previous year, and this is more likely to occur whilst located outside. Amelioration of EDS may assist in improving functional outcomes among these individuals by reducing the risk for falls.

## Background

Incidence of sleep-related problems are observed to increase with age [1], and a reduction in both the quality and quantity of nocturnal sleep and an increase in daytime symptoms are often considered a normal aspect of the aging process [2]. Excessive daytime sleepiness (EDS) is a common complaint among older adults, with research indicating that approximately 15 % of those aged 60years and older experience these symptoms [1]. The aetiology of EDS is multifactorial, particularly among older adults, and has been associated with individual markers of underlying sleep pathology, such as cases of obstructive sleep apnoea (OSA) [3]. These symptoms may similarly be associated with a number of medical or psychiatric conditions [4], or a number health or lifestyle factors, such as physical inactivity [5], that overlap with the risks for osteoporosis.

Among older individuals, EDS has been consistently and independently associated with an increased risk profile for several adverse health outcomes, such as reduced functional outcomes [6], depressive illness [7], deficits in cognitive abilities [8], as well as a two-fold increased risk for falls [9]. Research has suggested that approximately 30 % of older adults report sustaining one or more falls per year [10], and as many as 10-20 % of these incidents are associated with moderate to severe injury, such as fractures or severe head trauma [11]. Local estimates have suggested that one-tenth of time spent in hospital for those aged 65+ years is directly attributable to an injurious fall [12], and that these incidences account for the largest proportion of hospital admission for injuries among this age cohort [13]. Most hip fractures result from falls, the majority of which are treated in hospital [14, 15]. Thus, falls among older adults constitute a significant health burden and account for a large proportion of injury-related deaths in Australia [12].

Although a few studies have reported an association between EDS and increased risk of falls among older adults, there is currently insufficient information regarding the nature of the fall and degree of disability incurred as result of the fall. Specifically, there is currently little detailed information regarding details such as the location, circumstances and consequences surrounding the fall with regard to EDS. Moreover, there is inadequate collation of data pertaining to the relative contribution of factors such as concurrent medication use and other health behaviours on this association. Given both the high frequency of EDS and the health burden associated with falls in older adults, direct assessment of the relationship between these factors may assist in identifying possible modifiable factors, and thus, substantially improve primary preventative strategies for falls in these populations. Therefore, the aim of the current study is to describe the association between EDS and falls among a population-based sample of older adults.

## Methods

### Participants

This cross-sectional study examined men and women aged ≥60years who were enrolled in the Geelong Osteoporosis Study (GOS). The GOS is a large, population-based research project conducted in South-Eastern Australia. Population characteristics of the Barwon Statistical Division are comparable with national levels in terms of age, income, education level and marital status for each census taken in the years 1996, 2001 and 2006 [16]. Participants were randomly recruited using the Commonwealth electoral rolls for the region as a sampling frame. Both men and women were recruited utilizing an age-stratified sampling method.

Between the years 1993 and 1997, 1938 eligible women were randomly selected for inclusion into the study and 1494 agreed, representing 77.1 % participation [16]. At the 10-year follow up (2004-2008), 881 women returned for assessment (82.1 % retention rate). Of the total 881 women who participated in the 10-year follow up and who were aged ≥60 years (n = 453), participants whom complete sleep (n = 23), or anthropometry (weight and/or height) (n = 38) data were not available were excluded from analyses, resulting in a total of 367 eligible women aged 60-93 years.

Between the years 2001 and 2006, 3273 eligible men were randomly selected for inclusion into the study and 1540 agreed, representing 77.1 % participation [16]. Of those who were recruited, 978 men have since returned for follow-up (response 81.0 %). Of the total 978 men who participated in the 5-year follow up, participants who were aged <60 years (n = 479) for whom sleep data (n = 33), or anthropometry data (n =15) were not available were excluded from analyses, resulting in a total of 451 eligible men aged between 60-92 years.

Comprehensive descriptions of the male and female GOS cohorts and the related recruitment procedures can be found elsewhere [16].

This study was conducted with the approval of Barwon Health Human Research Ethics Committee, and written informed consent was obtained from each participant.

### Excessive daytime sleepiness (EDS)

EDS was assessed using the Epworth Sleepiness Scale (ESS) [17]. The ESS is a 4-point Likert-style questionnaire designed to assess participants’ average sleep propensity and likelihood of dozing among a number of hypothetically proposed activities, which include both active and soporific situations. Psychometric assessment of the ESS have demonstrated good overall internal consistency (Cronbach’s alpha = 0.88, *p* < 0.001) and test-retest reliability (Pearson’s r = 0.82, *p* < 0.001) [18], and has demonstrated good internal consistency, reliability and construct validity among older, community-dwelling adults [19, 20]. Although there is currently no universally accepted cut-off range for the ESS, similar studies have applied a cut-off of ≥10 to indicate EDS [21].

### Falls

Fall history was determined via a self-report questionnaire for both sexes. Falls were defined as an instance ‘when you suddenly find yourself on the ground, without intending to get there, after you were either in a lying, sitting or standing position’. Participants were classified as having had a fall if they reported one or more falls in the 12-month period prior to the time of assessment. Details of the fall(s) included the location of the fall, how the fall occurred, if the fall occurred from greater than standing height, whether the fall resulted in injury, and whether treatment was sought post-fall. Details of the fall(s) including the location and how the fall occurred were obtained as open-ended questions, and were transformed into categorical responses for analyses. Fall description was categorised using similar criteria used by comparable studies [22]. Responses that did not align with the descriptive categories were listed as ‘other’ (example includes response of ‘climbing on object’). Fall location was transformed into location-specific responses, which included ‘inside’ and ‘outside’.

A falls risk score was also obtained for male participants only using criteria outlined by Cwikel and colleagues (1998) [23]. The elderly fall screening test (EFST) consists of five items, in which a positive response to each item is counted as one point. Three items in the EFST refer to falls history which included: (1) number of falls; (2) falls resulting in injury; and (3) reporting ‘near falls’ occasionally or often and clinical assessments included: (4) measured walking speed (taking longer than 10 sec to cover 5 m); and (5) evidence of an unsteady or uneven gait. The risk profile has a total range of 0 (low risk) to 5 (high risk), with a positive indication to each item considered to equal one point. Total scores of >2 were considered as a high falls risk [23]. Using these criteria for statistical analysis, men were classified according to falls risk as having either a low or high risk. The EFST has been validated previously using a community-based sample of functional adults aged 60 + years [23]. Independent assessment of the EFST describes the measure as having good sensitivity and specificity (93 % and 78 %, respectively) [24].

### Lifestyle and heath factors

Anthropometric measurements were recorded objectively. Height and weight were measured to the nearest ±0.1cm and ±0.1kg, respectively. Body mass index (BMI) was calculated as weight/height squared (kg/m^2^). Waist (minimum circumference between the margin of the lower rib and iliac crest) and hip (maximal gluteal) circumference were measured using a narrow anthropometric tape measure. Participants were classified as centrally obese if they reported a waist measurement of ≥102 cm (men) or >88cm (women) [25].

Information regarding alcohol consumption and daily energy intake were obtained from the Cancer Council food frequency questionnaire [26]. Daily alcohol intake was expressed as gram intake per day (g/day), and energy intake was assessed as kilojoule per day (kJ/day). Level of mobility was assessed via a self-report questionnaire and transformed into a binary variable. Participants were classified as ‘active’ if they indicated a positive response to engaging in ‘moving, walking and working energetically and participating in vigorous exercise’; alternatively, participants were classified as sedentary. Current use of a mobility aid was assessed via self-report. Nocturia was assessed via self-report: participants indicated if they ‘often rose at night to use the toilet’ and how many times, on average, they needed the toilet during the night. Frequency responses were pooled into the categories 0-1, ≥2 times/night. Cases of nocturia were identified as those who used the toilet ≥2 times/night. Similar methods have been employed when assessing nocturia among samples of community-dwelling older adults [27]. Self-reported hours sleep per night was assessed as both a continuous and catagorised factor. Short sleep duration was defined as <6 hours/night, average sleep duration was 6-9 hours/night, and long sleep duration was classified as >9 hours/night. Similar methods have been employed to characterise sleep duration among comparable population-based studies [28]. Current use of tobacco smoking and medication use (sedatives/hypnotics and antidepressants) were documented by questionnaire. The presence of diabetes was identified by fasting plasma glucose >7.0mmol/L and/or positive self-reported diagnosis and/or use of insulin and/or hypoglycemic agents.

The presence of current depressive illnesses were assessed using the Structured Clinical Interview for DSM-IV-TR Research Version, Non-patient edition (SCID-I/NP) [29]. The use of this assessment tool allowed for the identification of current depressive disorders including: Major Depressive Disorder (MDD), bipolar disorder, dysthymia, minor depression, substance -induced mood disorder, and mood disorders due to a general medical condition.

Socio-economic status (SES) was assessed by cross-referencing the residential addresses of each participant to the Australian Bureau of Statistics (ABS) 2006 Census data; from which Socio-Economic Index for Areas index values were obtained. SEIFA values were applied to obtain an aggregated Index of Relative Socio-Economic Advantage and Disadvantage. Participants were categorised into quintiles of SES based on the BSD, where quintile 1 represented the most disadvantaged group, and quintile 5 represented the most advantaged.

### Statistical analyses

Univariate analyses were initially performed to assess the characteristics and associated lifestyle and health factors of those with and without EDS. Differences in characteristic data between those with and without EDS were analysed using a *t*-test for parametric continuous data, Mann-Whitney U for non-parametric continuous variables, χ^2^ analysis for discrete variables and Fisher’s Exact Test where expected cell sizes were less than five. EDS (yes/no) was applied as the exposure variable and differences among those with and without EDS in regard to fall history were tested using multivariate logistic regression models. Variables which were significantly associated with EDS in univariate analyses were added simultaneously to multivariable regression models. Interaction terms were checked to identify effect modification. Significance was set at p < 0.05. Statistical analyses were completed using Minitab (Version 16; Minitab, State College Pa).

## Results

### Women

#### Characteristics

Characteristic data for women with and without EDS are presented in Table 1. A total of 50 (13.6 %) women reported EDS. No differences were detected in regard to age, weight, height, BMI or waist circumference between groups.

**Table 1:** Characteristics of men and women, with and without EDS*

	Women				Men			
	All	No EDS	EDS*	*p*	All	No EDS	EDS*	*p*
	N = 367	n = 317	n = 50		N = 451	n = 379	n = 72	
Age	71.7 (65.1-78.5)	71.3 (65.2-78.4)	73.7 (63.6-80.1)	0.51	73.0 (66.3-80.3)	72.3 (65.6-79.2)	78.1 (69.4-85.1)	<**0.01**
Height (cm)	158.6 (154.0-162.8)	158.9 (154.0-162.7)	157.6 (154.0-163.1)	0.81	172.8 (167.8-177.0)	173.1 (168.0-177.1)	171.2 (167.1-175.1)	**0.05**
Weight (kg)	68.8 (60.5-78.2)	67.7 (60.1-76.8)	71.1 (64.4-82.6)	0.08	81.2 (73.7-91.7)	81.2 (73.8-91.2)	81.2 (72.1-93.3)	0.90
BMI (kg/m^2^)	27.4 (24.1-31.1)	27.0 (24.1-30.8)	28.6 (24.7-33.6)	0.09	28.0 (25.2-30.0)	27.5 (25.2-29.9)	27.9 (25.2-31.0)	0.27
Waist circumference^1^								
≥102 cm (men)±	-	-	-		202 (45.0 %)	164 (43.4 %)	38 (53.5 %)	0.12
≥88 cm (women)	211 (58.9 %)	178 (57.8 %)	33 (66.0 %)	0.27				
*Lifestyle*/*health factors*								
Reported at least one fall	118 (32.2 %)	94 (29.7 %)	24 (48.0 %)	**0.01**	94 (20.8 %)	75 (19.8 %)	19 (26.4 %)	0.21
≥2 falls	22 (6.0 %)	16 (5.1 %)	6 (12.0 %)	**0.05**				
Hours’ sleep (total)†	7.0 (6.0-8.0)	7.0 (6.0-8.0)	7.0 (6.0-8.0)	0.12	7 (6.0-8.0)	7.5 (6.0-8.0)	7.0 (5.5-8.0)	<**0.01**
Hours’ sleep/night †				0.26				**0.01**
(<6hr)	79 (21.8 %)	70 (22.4 %)	9 (18.0 %)		78 (17.3 %)	57 (15.0 %)	21 (29.2 %)	
(6-9hr)	237 (65.3 %)	206 (65.8 %)	31 (62.0 %)		330 (73.1 %)	283 (74.7 %)	47 (65.3 %)	
(>9 + hr)	47 (13.0 %)	37 (11.8 %)	10 (20.0 %)		43 (9.5 %)	39 (10.3 %)	4 (5.6 %)	
Use of walking aid	52 (14.5 %)	37 (11.8 %)	15 (30.0 %)	<**0.01**	48 (10.6 %)	30 (7.9 %)	18 (25.0 %)	<**0.001**
Nocturia	113 (30.8 %)	91 (28.7 %)	22 (44.0 %)	**0.03**	142 (31.5 %)	108 (28.5 %)	34 (47.2 %)	<**0.01**
Smoking (current)	26 (7.1 %)	20 (6.3 %)	6 (12.0 %)	0.14	28 (6.1 %)	27 (7.1 %)	1 (1.4 %)	0.07
Physically active	225 (61.3 %)	198 (62.5 %)	27 (54.0 %)	0.25	294 (65.2 %)	251 (66.2 %)	43 (60.0 %)	0.30
Alcohol intake (g/d)	0.9 (0.0-8.4)	0.9 (0.0-8.8)	0.4 (0.0-3.7)	0.36	9.3 (1.0-25.1)	10.5 (1.0-25.6)	5.5 (0.2-20.2)	0.11
Energy intake (kJ/d)	6165 (4995-7701)	6065 (4991-7576)	6730 (5230-8246)	0.07	355.2 (32.2- 825.8)	370.2 (36.9-837.0)	230.8 (7.1-731.1)	0.12
Sedative/hypnotic medication use (current)	21 (5.7 %)	19 (6.0 %)	2 (4.0 %)	0.75	8 (1.8 %)	8 (2.1 %)	0 (-)	0.21
Antidepressant medication use (current)	58 (15.8 %)	46 (14.5 %)	12 (24.0 %)	0.09	36 (8.0 %)	27 (7.1 %)	9 (12.5 %)	0.12
Mood disorder (current)	25 (6.9 %)	21 (6.7 %)	4.0 (8.0 %)	0.80	8 (1.8 %)	4 (1.1 %)	4 (5.6 %)	**0.03**
Diabetic status	26 (7.1 %)	16 (5.1 %)	10 (20.0 %)	<**0.001**	34 (7.6 %)	28 (7.4 %)	6 (8.3 %)	0.78
Socioeconomic status (current)				0.67				0.43
Quintile 1 (most disadvantaged)	60 (16.4 %)	53 (16.7 %)	7 (14.0 %)		85 (18.9 %)	73 (19.3 %)	12 (16.7 %)	
Quintile 2	68 (18.5 %)	55 (17.4 %)	13 (26.0 %)		94 (20.8 %)	83 (21.9 %)	11 (15.3 %)	
Quintile 3	103 (28.1 %)	89 (28.1 %)	14 (28.0 %)		91 (20.2 %)	78 (20.6 %)	13 (15.3 %)	
Quintile 4	66 (18.0 %)	58 (18.3 %)	8 (16.0 %)		87 (19.3 %)	69 (18.2 %)	18 (25.0 %)	
Quintile 5 (most advantaged)	70 (19.1 %)	62 (19.6 %)	8 (16.0 %)		94 (20.8 %)	76 (20.1 %)	18 (25.0 %)	

*denotes Epworth Sleepiness Scale (ESS) score ≥10

^1^as outlined by the International Diabetics Federations recommendations (2005) (see; methods)

±denoted n = 2 missing values

†denotes n = 4 missing values.

Values are given as median (interquartile range), mean (±standard deviation) or n (%)

No differences were detected between groups with regard to self-reported hours sleep (total) or hours sleep (categorised), current mood disorder status, alcohol intake, energy intake or SES. Compared to women without EDS, those with EDS were more likely have diabetes, report nocturia, and use a walking aid.

#### Falls

In total, 118 (32.2 %) of women reported at least one fall (range 1-4), and 24 (20.3 %) reported both EDS and fall history. A total of 22 women (6.0 %) reported ≥2 falls. Of the women who reported at least one fall, 14 (11.8 %) reported that the fall occurred greater than from standing height, and 80 (67.8 %) reported sustaining an injury as a result. Of those who sustained injuries, 55 (68.8 %) reported a soft tissue injury only (such as bruise or sprain) and 25 (31.3 %) reported a fracture as a result of the fall. No differences were noted between women with and without EDS in regard to the type of fall-related injury.

Falls were reported to occur most frequently as a result of tripping (44.3 %), slipping (15 %), other (15 %), loss of support/surface structure (10.6 %), knocked over (6.2 %), loss of balance (5.3 %) and legs giving way (3.5 %) (data not shown). Numbers were too small to detect differences in cause of fall among those with and without EDS.

Women with EDS were more likely to fall outside (34.8 % inside vs. 65.2 % outside), and women without EDS were more likely to fall inside (59.6 % inside vs. 40.4 % outside) (*p* = 0.03).

EDS was associated with an increased risk of reporting a fall in the previous 12 months (unadjusted OR = 2.19, 95 % CI 1.20-4.01, p = 0.01), and this was attenuated following adjustment for use of a walking aid, cases of nocturia and antidepressant medication use (adjusted OR = 2.54, 95 % CI 1.24-5.216, p = 0.01). These findings were independent of age and diabetic status (Table 2).

**Table 2:** Odds Ratios (OR) and 95 % Confidence Intervals (CI) for the association between excessive daytime sleepiness (EDS)* and falls for women^1^ and men^2^, unadjusted and fully adjusted models

	OR	95 % CI	p-value
Women			
*Unadjusted*			
EDS	2.19	1.20, 4.01	**0.01**
*Adjusted*			
EDS	2.54	1.24, 5.21	**0.01**
Age	0.99	0.96, 1.03	0.80
Walking aid	2.20	1.00, 4.18	**0.05**
Nocturia	1.56	1.01, 2.80	**0.05**
Diabetic status	0.88	0.34, 2.01	0.70
Antidepressant medication use	4.54	2.34, 8.83	**0.001**
EDS*Antidepressant medication use	0.19	0.04, 0.85	**0.03**
Men			
*Unadjusted*			
EDS	1.78	0.04, 1.19	**0.06**
*Adjusted*			
EDS	1.26	0.65, 2.52	0.48
Age	1.10	1.03, 1.10	<**0.01**
Height	1.00	1.00, 1.00	0.74
Hours sleep/night	0.92	0.77, 1.09	0.32
Nocturia	1.05	0.60, 1.85	0.85
Mood disorder (current)	1.98	0.36, 10.75	0.43

*Denotes Epworth Sleepiness Scale (ESS) score ≥10

^1^Self-reported falls in the previous 12 months

^2^Elderly Falls Screening Test (EFST) scores (referent value EFST score <2)

Significant values are indicated by bold font

Initial multivariate modelling revealed antidepressant use (current) as an effect modifier (*p* < .001 for the interaction term). Data were thus stratified by exposure to antidepressant medication, and a relationship among antidepressant non-users, EDS and self-reported falls in the previous 12 months was noted (unadjusted OR = 2.74, 95 % CI 1.37-5.49, p < 0.01). The relationship between EDS and falls was further sustained following adjustment for nocturia (adjusted OR = 2.63, 95 % CI 1.31-5.30, p < 0.01). These findings were independent of smoking status, BMI, alcohol intake, sedative medication use and use of a walking aid. No relationship was observed between EDS and falls for antidepressant users.

### Men

#### Characteristics

Characteristic data for those men with and without EDS is presented in Table 1. A total of 72 (16.0 %) men reported EDS. Those men who reported EDS were older than men without EDS (*p* < 0.01). No differences were noted between groups in regard to weight, BMI or waist circumference.

Compared to men without EDS, men with EDS were more likely to use a walking aid, were more likely to report nocturia, current depressive illness, fewer total hours of sleep/night, and <6 hours sleep/night (all *p* < 0.05). No differences were detected in regard to smoking status, physical activity level, alcohol intake, energy intake, diabetic status, SES, or medication use.

#### Falls

In total, 94 (20.8 %) men reported any fall (range 1-3) during the previous year, and 19 (20.2 %) reported both EDS and a fall. Multiple falls were reported by 7 (2.0 %) men. Of those men who reported falls, 25 (26.6 %) reported that the fall occurred from greater than standing height, and 47 (50.0 %) reported sustaining an injury as a result of the fall. Of the men who reported sustaining an injury, 38 (80.9 %) reported a soft-tissue injury and 9 (19.1 %) reported a fracture. No differences were noted between men with and without EDS in regard to the type of fall-related injury.

Falls were reported to occur most frequently as a result of tripping (45.6 %), followed by other (32.2 %), slip or loss of support/surface structure (both 7.8 %), loss of balance (5.6 %), and from being knocked over (1.1 %).

No difference was noted between the fall location between those with and without EDS (data not shown).

No differences were detected for those men with and without EDS in regard to self-reported falls in the previous 12 months. As a result, a multivariable model was not developed for this outcome.

EFST scores displayed a range of 0-4. A total of 73 (16.2 %) men met criteria for a high falls risk. A trend towards significance was noted between men who reported EDS and the likelihood of having a high falls risk (p = 0.06), however, age explained this relationship (age adjusted OR = 2.20, 95 % CI 1.03-1.10). These findings were independent of height, hours sleep/night, cases of nocturia and mood disorder (current) (Table 2).

## Discussion

Over one tenth of men and women aged >60years assessed in this study report clinically significant levels of EDS. The results from this cross-sectional study suggest that for women, EDS is associated with an approximate two-fold increased likelihood of reporting at least one fall during the previous year, independent of a number of confounding factors. Further, women with EDS were more likely to report a fall occurring whilst located outside, whereas women without EDS were more likely to report a fall whilst located inside. No association was observed for men with regard to EDS and falls history; however, a trend towards significance was noted between EDS and an increased risk profile for falls as assessed by the EFST. Both men and women reported falls occurring most frequently as a result of tripping and indicated that soft-tissue injuries were the most common reported injury sustained as a result of the fall.

Over one-tenth of older men and women sampled in this study report EDS (16 % and 13.6 %, respectively), which is comparable to population-based prevalence we have cited previously [1], but higher than that cited by others [9]. Healthy, independent community-dwelling older adults often report lower rates of EDS than those who reside within aged-care facilities [30]. This may in part be attributed to typically higher levels of physical functioning and greater levels of independence among these individuals [31], as well as lower overall rates of peripheral factors often associated with EDS, such as disease comorbidity [32] and increased rates of polypharmacy [33]. We further report an overall fall prevalence for both men and women that is comparable to [34], but lower than some [35] of the previous research assessing falls among healthy, community-dwelling adults. Higher rates of falls are often observed among older adults living within aged care or assisted living facilities compared to those living within the community [36, 37]. Indeed, individuals living in long-term institutionalised care have as much as a threefold increased risk of reporting a fall, and a 10-25 % increased risk of sustaining injuries such as a fracture or laceration as a direct result of a fall [38]. Falls are often considered independent determinants of functional decline and worse disability outcomes among older community-dwelling adults [39]; and are often cited as the primary contributing factor for later admission to institutionalised care [38]. Community-based prevention strategies are therefore pivotal in the reduction of nursing home admissions among at-risk individuals.

The associations between EDS and falls among population-based samples of older adults have only been examined among a limited number of studies. Similar research conducted by Teo and colleagues [9] assessed the role of nocturnal sleep disturbance, cases of urinary incontinence and instances of EDS in regard to the relative risk for falls among a cohort of older Australian women. It was reported that those with EDS were more likely to report a fall than those who did not report EDS (univariate analysis), and that EDS was the strongest risk factor for reported falls after controlling for other recognised falls risk factors. We report similar increased odds for falls among women with EDS as those presented by Teo and colleagues [9]; moreover, we were able to address some limitations of this research by accounting for and assessing the relative contribution of a larger number of associated health and lifestyle factors, such as mobility levels and exposure to sedative/antidepressant medication. We report that the association between EDS and falls among women is observed only among non-users of antidepressant medications, however, and thus the role of this relationship in the expression of falls risk among affected individuals is unclear. It is possible that other drug classes, as well as drug interactions are contributing to these findings. As a comprehensive examination of medication classes and drug interactions were beyond the scope of this study, further research would benefit from direct and comprehensive assessment of the association between EDS, antidepressant and other medication use and falls risk.

Among men, no relationship was detected between EDS and falls, and only a trend towards significance was noted between EDS and falls risk as assessed by the EFST score. These findings, in part, mirrors previous research which has typically suggested a lower overall prevalence of falls among older men compared to women [34, 40], and reported that female gender represents a risk factor for falls [34]; however this finding is not universal [10]. Singular aspects of the EFST have been shown to be accurate predictors of falls risk in men, such as slow and/or unsteady or gait [41], however balance and/or gait assessments alone are not considered effective predictors of relative falls risk [42]. We acknowledge that we did not observe an association between EDS and falls, and suggest that this finding may, in part, be due to underreporting of falls by the male participants. Furthermore, as only a small proportion of men reported both a fall and EDS, we cannot exclude the possibility of a type 2 error. Future research would benefit from corroborating both objective and subjective fall records in order to recognise any possible sources of personal bias in responses.

We report that a greater proportion of women with EDS report a fall occurring whilst located outside. These findings may reflect the sample population; as activities such as gardening are often cited as the most frequently engaged form of physical activity among healthy older adults, particularly during the warmer months (i.e., summer/autumn) [43]. Further, this may in part provide explanations for the observation of a greater proportion of women with EDS reporting a fall whilst located outside; however, the direct mechanism driving this association is unknown. Indeed, other studies have noted no significant seasonal variation in fall incidence rates in the study region which is located in a temperate climate [44] . We did not have access to seasonal markers as part of this study, however, further research may benefit from disseminating seasonal correlations and patterns associated with fall location to better target at-risk individuals during these times. Additional research would benefit from tabulation of the amount of time spent indoors/outdoors, either in the form of a questionnaire or objective assessment of indoor/outdoor activity (such as Actigraphy monitoring) in order to evaluate this association in more detail.

Injuries resulting from falls represent a significant factor in hospitalization and subsequent functional decline among older adults [45]. Soft-tissue injuries were the most common injury sustained as a result of a fall for both men and women, and we report a higher proportion with these injuries than has reported elsewhere (e.g., [30]). It is possible that within our study, the methodology employed to classify sustained injuries was more inclusive of reported injuries. As the questionnaire used was an open-ended item, we were able to classify all responses into injury categories (soft-tissue, fracture etc.), thus possibly resulting in higher incidence. Prevention strategies aimed at older individuals, such as strength and/or gait training, reduction in tripping hazards, typically emphasise the risk-reduction of incidences occurred inside the home only. As we similarly report a high rate of falls occurring among women with EDS when situated outside, similar programs and/or education aimed at addressing possible risks and hazards among these locations may assist in reducing the number of falls and incurred injuries within these settings.

When considering the mechanistic pathways which may drive an association between sleepiness and falls, it is feasible to assume that diurnal disruptions characteristic of the aging process, such as a reduction in total sleep time, reduced sleep efficiency and increased sleep fragmentation may contribute to impairments in behavioural and cognitive functions similarly implicated in falls [46]. Coupled with increased rates of polypharmacy often observed among these age groups, it is also likely that these factors interact to contribute to and compound impaired levels of cognition; which is directly associated with increased falls occurrence and future falls risk. Indeed, reduced executive function capabilities, which can be impaired as a result of sleepiness [47] and often are associated with increased or excessive poly-medication use (such as sedatives, painkillers, cardiac medications etc.) [48]; have also been shown to contribute to falls among older men and women [49], and measures of these variables have demonstrated efficacy in predicting later falls [50]. Differences in mechanistic properties driving an association between EDS and falls between men and women may, in part, be due to differing sex-specific characteristic sleep and medical profiles. Among men, it is possible that both indirect (weight, neck circumference, BMI) and direct (snoring, nocturnal choking) factors associated with underlying OSA represents a more salient factor contributing to EDS and subsequent falls. Among women, it is possible that poor nocturnal sleep quality or instances of insomnia, which is more common among older women [51] contribute to EDS, and that subsequent compensatory mechanisms (taking sleep-enhancing medication) contribute to reported falls as a function of reduced alertness and attention during waking hours. Therefore, it is proposed that both the transient and chronic effects of sleep loss or poor sleep, compounded by the effects of poor-health and disease and subsequent compensatory measures (such as increased medication use), which typically differ between sexes, may directly affect neurocognitive functions commonly implicated in falls, such as alertness, attention and executive functioning. As a comprehensive examination of many of these domains were beyond the scope of this study, further assessment of the association between EDS, medication use, objective sleep factors and falls risk factors may assist in further describing these mechanistic pathways.

A notable strength of the current study included utilizing a large, representative population-based sample of older men and women. Due to the study design, we were able to obtain a representative sample of elders, and thus we overcame the limitations of previous research which often utilise techniques that are sensitive to volunteer bias [22, 30]. Furthermore, the use of population-based older adults in the current study addresses the limitations of previous studies which have often utilised sample populations solely drawn from assisted living or retirement facilities [see [3]]. Similarly, we were able to obtain and collate detailed information regarding the nature and circumstances of the reported falls, such as information pertaining to the number of falls, the fall location, the reason for falls, and whether any injuries occurred as a result of the fall; therefore highlighting some possible areas of intervention.

Although we present a significant association between the presence of EDS and falls in women, it must be acknowledged that inferences regarding the directionality of the relationship cannot be made due to the cross-sectional study design. We assessed falls history using a retrospective questionnaire design over the past 12 month period; a method which has been employed by previous population-based studies of older adults [9,34]. Although this technique is useful with regard to assessing approximations of the burden of disease, poor or inaccurate fall recollection over this time period cannot be excluded. Despite this, meta analyses have shown good overall specificity (91-95 %) and adequate sensitivity (80-89 %) of self-reported recollection of falls occurring in the preceding 12 months among healthy older individuals [52], and we report similar rates to previous studies [9]. Due to the small number of individuals who reported multiple falls, we did not assess this association in detail with regard to EDS. Further research would benefit from investigating whether symptoms of sleepiness contribute to reporting multiple falls, and whether it represents an independent risk factor. Further, some research has highlighted the limitations of the ESS as a measure of daytime somnolence among some groups of older adults due to individuals being unable to fully complete the questionnaire despite reporting symptoms of EDS [53]. However, assessments of the reliability of the ESS among these individuals are typically conducted on cohorts of clinical groups from selective hospital outpatient departments [53], and therefore the same issues may not be present in all adults. Lastly, as we did not explicitly assess instances of sleep disordered breathing such as OSA, we cannot exclude that this may have contributed to the current findings. Despite this, previous studies have found that among community dwelling adults, a weak correlation exists between instances of sleep disordered breathing and daytime impairment [54].

## Conclusion

These data suggest that among this population-based group of older adults, EDS is associated with an increased incidence of falls for women, independent of a number of associated health and lifestyle factors, and that these falls are most likely to occur whilst located outside. For men, a trend towards significance was noted for the association between EDS and an increased falls risk profile. As EDS is a common symptom among older adults, and falls are often considered a precipitating factor of assisted living admission, amelioration of these symptoms may improve functional outcomes for these individuals and preserve independent living status and thus reduce the risk for morbidity among these individuals. Future research would benefit from the inclusion of more comprehensive assessments of specific sleep pathology in order to explicate their role in the reported relationship.
